# Alternative sigma factor σ^H ^activates competence gene expression in *Lactobacillus sakei*

**DOI:** 10.1186/1471-2180-12-32

**Published:** 2012-03-12

**Authors:** Solveig Schmid, Claudia Bevilacqua, Anne-Marie Crutz-Le Coq

**Affiliations:** 1 UMR1319 Micalis, INRA F-78350, Jouy-en-Josas, France; 2 UMR Micalis, AgroParisTech, INRA F-78350, Jouy-en-Josas, France; 3 UMR1313 Génétique Animale et Biologie Intégrative, plateforme ICE, INRA F-78350, Jouy-en-Josas, France; 4 Conceptus SAS, 50 avenue de Saint Cloud, F-78000 Versailles, France

## Abstract

**Background:**

Alternative sigma factors trigger various adaptive responses. *Lactobacillus sakei*, a non-sporulating meat-borne bacterium, carries an alternative sigma factor seemingly orthologous to σ^H ^of *Bacillus subtilis*, best known for its contribution to the initiation of a large starvation response ultimately leading to sporulation. As the role of σ^H^-like factors has been little studied in non-sporulating bacteria, we investigated the function of σ^H ^in *L. sakei*.

**Results:**

Transcription of *sigH *coding for σ^H ^was hardly affected by entry into stationary phase in our laboratory conditions. Twenty-five genes potentially regulated by σ^H ^in *L. sakei *23 K were revealed by genome-wide transcriptomic profiling of *sigH *overexpression and/or quantitative PCR analysis. More than half of them are involved in the synthesis of a DNA uptake machinery linked to genetic competence, and in DNA metabolism; however, σ^H ^overproduction did not allow detectable genetic transformation. σ^H ^was found to be conserved in the *L. sakei *species.

**Conclusion:**

Our results are indicative of the existence of a genetic competence state activated by σ^H ^in *L. sakei*, and sustain the hypothesis that σ^H^-like factors in non sporulating Firmicutes share this common function with the well-known ComX of naturally transformable streptococci.

## Background

Sigma factors are subunits of the RNA polymerase complex responsible for specific recognition and melting of promoter DNA, which enable the polymerase to initiate transcription. All eubacteria of known genome sequence code for at least one sigma factor, called primary, housekeeping or vegetative, and most encode additional sigma factors. For example, *Streptomyces coelicolor *or *Sorangium cellulosum *carry as many as 60 to 80 predicted sigma factors [1,2]. These so-called alternative sigma factors may be induced or activated by specific environmental signals, and consequently redirect transcription by competitively associating with the core RNA polymerase. Alternative sigma factors have been shown to mediate various cellular responses linked to stress conditions, growth transitions or morphological changes and development [1].

Sigma factors are classified into two structurally and evolutionarily distinct superfamilies [3], σ^70 ^and σ^54^. Most eubacterial sigma factors belong to the σ^70 ^superfamily, which is further divided into four phylogenetic groups on the basis of protein structure and physiological function [1,4]. Group 1 comprises the housekeeping sigma factors. Group 2 is close to group 1 but accommodates non essential sigma factors, including the master regulator of general stress response in stationary phase, RpoS, as was well characterized in *Escherichia coli*. Sigma factors in group 3 are phylogenetically diverse, and regulate major cellular functions such as sporulation, motility, heat-shock or general stress response. Group 4, known as the extracytoplasmic function (ECF) subfamily, has been distinguished more recently. It comprises highly diverged sigma factors mainly involved in responses to extracytoplasmic stimuli, which may affect the correct folding of envelope proteins. These factors typically contain only domains refgrped to as 2 and 4, involved in core polymerase binding and promoter DNA recognition and melting [3], with a spacer domain of less than 50 residues [2]. However, due to the high divergence across sigma factors, their classification in the previously identified phylogenetic groups may need to be revised, and new cellular functions controlled by sigma factors may be discovered [4].

Our research concerns a putative σ^H ^factor in the lactic acid bacterium *Lactobacillus sakei*. The closest characterized homolog is the σ^H ^of *Bacillus subtilis *(σ_Bsu_^H^), encoded by *sigH *(formerly s*po0H*), which is best-known for its role in initiating sporulation, an ultimate differentiation response to starvation. σ_Bsu_^H ^directs transcription of genes involved in polar septum formation and provokes induction of several regulator genes that in turn affect expression of signaling pathways or turn on pathways for endospore engulfment (e.g. *via *the σ^F ^sigma factor) [5,6]. σ_Bsu_^H ^is also associated with genetic competence, which enables the uptake of exogenous DNA and its assimilation as new genetic information, leading to natural genetic transformation. This transient state occurs in about 10% of the cells as part of the same nutrient depletion response as sporulation. σ_Bsu_^H ^increases expression of one of the two peptide pheromones needed for optimal activation of the master regulator of the competence pathway ComK [7,8]. While σ_Bsu_^H ^is essential for initiating sporulation, its absence reduces, but does not abolish transformation (efficiency is decreased by ~16-fold) [9]. The whole decision-making pathway leading to sporulation or competence is an elaborate signal transduction network relying on multiple partners [7,10]. In addition, σ_Bsu_^H ^reportedly affects expression of about 10% of the genome and was proposed to be involved in the growth transition to stationary phase [5].

The position of σ_Bsu_^H ^in the tree of σ^70^-type sigma factors is unclear. It exhibits structural characteristics similar to ECF sigma factors (group 4), yet phylogenetic analyses placed it between groups 3 and 4 [2,4,11]. Unexpectedly, database and phylogenetic analyses revealed that σ^H^-like gene products are largely distributed in various members of the Firmicutes, sporulating or not [11,12]. Interestingly, σ^H^-like factors appear to be more divergent across non-sporulating bacteria than in sporulating bacteria [12]. At the same time, structural elements similar to the conserved Gram-positive DNA uptake machinery appeared to be encoded in the genome in members of the Firmicutes not known for being naturally transformable, suggesting that this capacity may be more widespread than previously expected [12-14]. Two factors, classified in a single large σ^H^-family of sigma factors by Morikawa *et al. *[12], are directly involved in transcription of competence genes in non-sporulating bacteria: the well-known ComX of naturally transformable streptococci [15], and the product of the so-called *sigH *gene of *Staphylococcus aureus*, a species which has not yet been shown to be transformable [12]. These observations suggested the link between σ^H^-like factors and genetic competence in non sporulating Firmicutes [12].

*L. sakei *belongs to the microbiota that develops on meats under storage, especially during vacuum packing. It is largely used as a starter for the manufacture of fermented sausages in Western Europe and its potential use in meat product biopreservation is currently under study [16-18]. Survival of *L. sakei *ranges from one day in aerated chemically defined liquid medium, to a few months in dry sausages, although little is known about the factors determining its stability. The existence in *L. sakei *of *sigH*_Lsa_, an apparent *sigH*_Bsu _ortholog, led us to identify the gene set regulated by σ_Lsa_^H^, and to determine whether and how this regulator is implicated in competence and stationary phase survival. A strain allowing experimental *sigH*_Lsa _induction was constructed, and used in a genome-wide microarray study. Genes activated by *sigH*_Lsa _overexpression appeared mainly involved in genetic competence, although we could not obtain evidence for natural transformation. This study provides further suggestive evidence that the conserved role of the σ^H^-like sigma factors in non-sporulating Firmicutes is to activate competence gene expression.

## Results and discussion

### Identification of *sigH *in the genome of *L. sakei *and other lactobacilli

**Figure 1:**
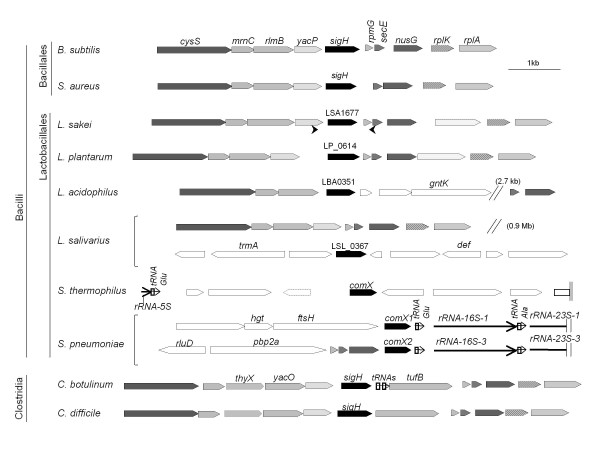
**Genetic context of *sigH *and σ^H^-like genes in members of the Firmicutes**. The figure was generated using Microbes on line facilities http://www.microbesonline.org. Similarly filled arrows represent homologous CDSs. White arrows indicate CDSs without counterpart. Pseudogene is indicated by a dotted outline. RNA-encoding genes are represented by thin arrows. Two loci are shown for *L. salivarius*, one demonstrating the absence of a *sigH *counterpart in the same genetic context as *B. subtilis *and the other, at a distance of 0.9 Mb, showing the *sigH *homologous gene in its genetic context. Two loci are also shown for *S. pneumoniae*, which possesses two identical copies of *comX*. Positions of primers AML50 (upstream) and AML58 (downstream) are indicated by small arrows under the *L. sakei *s*igH *locus. Species are represented by the same strains as listed in Figure 2.

Automatic annotation of the *L. sakei *23 K genome [16] identified LSA1677 as a coding sequence (CDS) of a putative alternative sigma factor of the σ^70 ^superfamily. It belongs to COG1595 (E-value of 7e^-6^), which comprises both ECF-type sigma factors (*E. coli *RpoE homologs) and σ^H ^of *B. subtilis*, and thus reflects the reported structural proximity between ECF sigma factors and σ_Bsu_^H ^[2,4,11]. The conserved genetic context of the *L. sakei *LSA1677 locus and the *B. subtilis sigH *locus, and more generally the local synteny between several members of the Firmicutes (Figure 1), revealed that LSA1677 and *sigH*_Bsu _are likely orthologous genes, belonging to a widespread family in the Firmicutes.

Nevertheless, the locus comprising σ^H^-like gene may have experienced genetic rearrangements across the different genera and also among species of the same genus (Figure 1). Moreover, the σ^H^-like gene location seems to be variable in members of the Firmicutes, especially in the *Lactobacillales *(Figure 1). A putative σ^H^-like gene is not found at the same location in *Lactobacillus salivarius *as in *L. sakei *(locus *cysS*-*nusG*). Likewise, the location of the unique gene for the ComX factor differs in the naturally competent *Streptococcus thermophilus *LMD9 from those of each of the identical *comX *copies in *S. pneumoniae *R6, in which both copies are adjacent to a tRNA gene and ribosomal operons.

**Figure 2:**
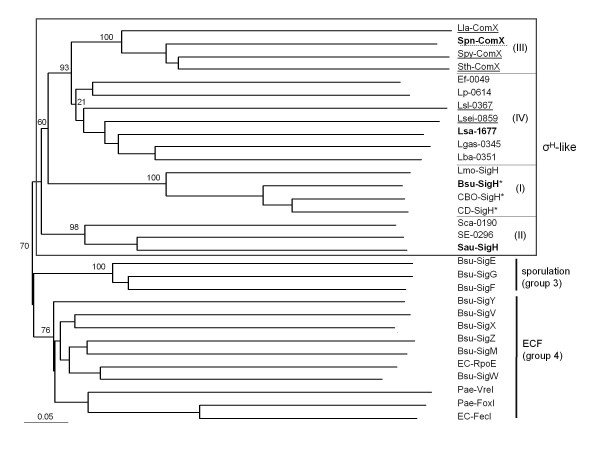
**Clustering of selected σ^70^-superfamily of sigma factors**. The unrooted tree resulted from a multiple alignment over the whole aa sequence length of σ^H^-like factors and known sigma factors from group 3 (sporulation factors of *B. subtilis*) and group 4 (ECF factors from *B. subtilis *and Gram-negative bacteria). The multiple alignment was generated using clustalX [19], by introducing first the shortest sequences to ensure a correct alignment of the conserved regions. The tree was drawn with NJplot http://pbil.univ-lyon1.fr/software/njplot.html. Bootstrap values (number of seeds: 1000, number of trials: 100) are indicated for the upper branches. Evolutionary distance is represented by branch length (scale at the bottom). Groups of σ^H^-like factors were numbered as previously reported [12] and a fourth group (IV) was added by our analysis. Bsu, *Bacillus subtilis *168; EC, *E. coli *K-12 substr. MG1655; Pae, *Pseudomonas aeruginosa *PAO1; Ef, *Enteroccocus faecalis *V583; Lla, *Lactococcus lactis *Il1403; Lmo, *Listeria monocytogenes *EGD-e; Genus *Clostridium*: CBO, *C. botulinum *A ATCC3502; CP, *C. difficile *630. Genus *Lactobacillus*: Lba, *L. acidophilus *NCFM; Lsei, *L. casei *ATCC334; Lgas, *L. gasseri *ATCC 33323; Lp, *L. plantarum *WCFS1; Lsa, *L. sakei *23 K, Lsl, *L. salivarius *UCC118; Lac, *L. acidophilus *NCFM. Genus *Staphylococcus*: Sau, *S. aureus *N315; Sca, *S. carnosus *TM300; SE, *S. epidermidis *ATCC 12228. Genus *Streptococcus*: Spn, *S. pneumoniae *R6; Spy, *S. pyogenes *ATCC 10782; Sth, *S. thermophilus *LMD-9. Names of gene products or locus tags are indicated. σ^H^-like sigma factors which belong to sporulating bacteria are indicated with an asterisk; those encoded by a gene not located at a similar locus to *sigH*_Bsu _are underlined (dashed line for the particular case of *S. pneumoniae*, see Figure 1). The best studied σ^H^-like sigma factor for each group is in bold type.

Although the genetic context of the σ^H^-like locus is very well conserved between *L. sakei *and *Lactobacillus plantarum*, the two σ^H^-like proteins share only 29% amino acid (aa) identity. Indeed, the level of inter-species aa identity of σ^H^-like gene products across the genus *Lactobacillus *is low (e.g., < 20% between *L. plantarum *WCFS1 and *L. jensenii *208-1 to 67% between *L. helveticus *DPC4571 and *L. crispatus *MV1AUS). The LSA1677 gene product shares weak aa identity with the σ^H ^factors of *B. subtilis *(24%) and *S. aureus *(21%), as well as 22% aa identity with ComX of *S. pneumoniae *(see Additional file 1: Alignment of four σ^H^-group sigma factors). Due to the high sequence divergence between sigma factors, a robust phylogeny is difficult to achieve. Tentative clustering of σ^H^-like sigma factors (Figure 2), also including sporulation and known ECF sigma factors of *B. subtilis*, separates σ_Bsu_^H ^from the other sigma factors in that species and argues for the existence of a σ^H^-type family in Firmicutes [12]. σ^H^-like factors appear to form groups mostly congruent with the genus phylogeny, irrespective of the location of the relevant gene in the genomes (Figure 2). The σ^H^-like sigma factors of lactobacilli added a fourth group to the three previously reported groups (whose type factors are σ_Bsu_^H^, σ^H^-like of staphylococci and ComX of streptococci) [12]. LSA1677 exhibits the characteristics of a σ^H^-like factor encoding gene and, in view of the conserved genetic context between *L. sakei *and *B. subtilis*, was called *sigH*. Note that the name *sigX *has been chosen for recently annotated genomes of *Lactobacillales*. Although the name SigX is more appropriate than ComX for a sigma factor, it adds confusion with the existing SigX sigma factor of *B. subtilis*, which is not the equivalent of σ^H^. This certainly calls for a unified nomenclature of sigma factors in Firmicutes.

### Conservation of *sigH *genes in the *L. sakei *species

**Figure 3:**
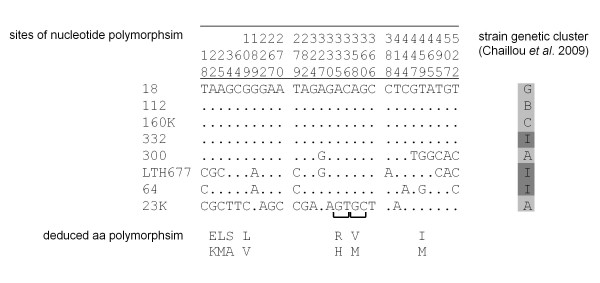
**Polymorphic nucleotide sites of *sigH *sequences in *L. sakei***. The entire CDS sequence (561 nt) was analyzed with MEGA software http://www.megasoftware.net/. Only nucleotide residues different from the upper line sequence are written. The site numbers at the top are in vertical format. Letter-code genetic cluster according to Chaillou *et al. *[20] is indicated for each strain and reported subspecies are shaded differently. Polymorphic deduced aa are indicated under the sequence.

We asked whether *sigH *genes were conserved among *L. sakei *isolates exhibiting a broad intraspecies diversity [50]. Based on the presence or absence of markers of the flexible gene pool, *L. sakei *isolates from various sources were previously classified into distinct genotypic clusters, possibly affiliated with two prevailing sub-species [20]. The 5' and 3' ends of the *sigH *gene were used as targets for PCR amplification of 17 isolates belonging to 9 of the 10 reported clusters of the species [20]. A unique fragment of the same size as that of strain 23 K was amplified for all samples, indicating that the gene is likely present in all tested isolates. Further analysis of the locus was undertaken for 7 of these strains distributed in 5 clusters. Amplification obtained with primers designed on the basis of the *L. sakei *23 K genome outside of *sigH *suggested that the genetic context is conserved in all these strains (see position of primers AML50 and AML58 in Figure 1). Polymorphism analysis of the *sigH *sequences brought additional information. As shown in Figure 3, 29 polymorphic sites were identified in the *sigH *CDS, of which only 9 were involved in 7 aa changes, mostly conservative. Thus, SigH function and coding gene location appear to be conserved in the *L. sakei *species.

*L. lactis *subspecies *lactis *and *cremoris *exhibit two *comX *allelic types whose nucleotide divergence is at most 27.5% [21]. In contrast, *sigH *divergence (4.5% maximum divergence) was incongruent with the previously reported genotypic classification of *L. sakei *strains [20], and its two proposed subspecies (Figure 3). This discrepancy may be explained either by a particular evolutionary history of that gene in *L. sakei *or by the possibility that the classification based on the flexible gene pool does not reflect the phylogenetic relationships between strains which remain to be established.

High nucleotide divergence between species, contrasted with generally higher conservation within species, was also observed for *sigH *loci in the genus *Staphylococcus *[22]. The reason for such high inter-species polymorphism is unknown. However, rapid evolution after species divergence rather than lateral gene transfer may be responsible, as the phylogeny of *sigH *genes was reported to be concordant with species phylogeny in staphylococci [22].

As reported in this paper, functional studies were further conducted on RV2002, a derivative of *L. sakei *strain 23 K, for which genome data is available, and in which the endogenous β-galactosidase encoding gene is inactivated, thus enabling the use of a *lacZ *reporter gene [23].

### Temporal transcription of *sigH*

**Figure 4:**
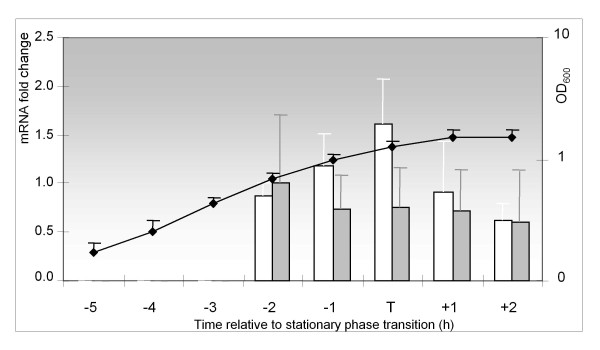
**Temporal transcription of *sigH***. Growth of RV2002 has been monitored by OD_600 _(right axis). Time is indicated in hours relative to the approximate transition to stationary phase (T). mRNAs levels of *ldh *(grey blocks) or *sigH *(white blocks) were measured by qPCR and expressed as fold change relative to an early-exponential calibrator sample (left axis). For *sigH*, results have been further normalized by *ldh *mRNA levels and expressed as *sigH*/*ldh *ratio. Error bars represent standard deviation. A fold change of 1 indicates a constant level of transcripts.

In *B. subtilis*, *sigH*_Bsu _transcription increases from mid-exponential to stationary phase [24]. We used quantitative PCR (qPCR) following reverse transcription to determine if *sigH*_Lsa _expression in *L. sakei *is also temporally regulated. *L. sakei *was cultivated in chemically defined medium (MCD) at 30°C and total RNA was extracted from cells 2 h after inoculation and every hour from 4 to 8 h. In these experiments, transition to stationary phase was observed between 5.5 and 7 h. Three genes, *ldh*, *gyrA *and *sigA*, were initially evaluated as candidate internal standards for qPCR, based on previously used standards in *Oenococcus oeni *[25]. We selected *ldh*, which showed the least variation of mRNA levels during growth (Figure 4). *sigH*_Lsa _mRNA levels were then quantified relative to the early-exponential condition (2 h) chosen to calibrate the measurements, and by normalizing with *ldh *mRNA. Results showed a slight increase (1.7 ± 0.3) of *sigH*_Lsa _transcripts around the transition to stationary phase (Figure 4). This transcription pattern is close to that reported for *B. subtilis*, for which *sigH*_Bsu _transcription reached a 3-fold increase peak 40 min before transition to stationary phase in sporulation medium [24]. Possibly, the observed level of *sigH*_Lsa _induction could be greater in other media and growth conditions. *sigH*_Bsu _repression during exponential growth phase relies on the transcriptional repressor AbrB, a major transition-state regulator in *B. subtilis *[24]. As no homolog of AbrB could be identified in *L. sakei*, we suspect that other regulatory circuit may be involved in controlling *sigH*_Lsa_. Interestingly, *S. aureus *s*igH*_Sau _transcription reportedly decreases 10-fold from early-exponential to stationary phase [26].

### Overexpression of σ^H^

The s*igH*_Lsa _gene was overexpressed as a means to reveal genes that it specifically regulates. *sigH*_Lsa _was placed under the control of the copper-inducible *L. sakei *promoter P_atkY_, present on plasmid pRV613 [27], and the resultant plasmid was introduced into RV2002 wild-type (WT) strain. The resulting strain, designated *sigH*(hy)*, thus has an additional expression-controlled copy of *sigH *and was compared to the equivalent WT strain harboring the pRV613 plasmid, in which P_atkY _controls *lacZ *(see additional file 2: Genotype of *L. sakei *strains affected in *sigH*). We anticipated that competence genes, found in the *L. sakei *genome and likely coding for a DNA uptake machinery [28], might be target genes for transcription by σ^H^-directed RNA polymerase (see additional file 3: Competence DNA uptake machinery of *B. subtilis *and comparison with *L. sakei*). We therefore used qPCR to monitor *sigH*_Lsa _and c*omEA *transcripts produced by cells at 1 h (the time needed to obtain ~15-fold induction of P_atkY_; [27]) and 4 h (corresponding roughly to the transition to stationary phase) after induction in mid-exponential phase, or without induction. Addition of CuSO_4 _to the strain harboring the control plasmid had no detectable effect on the amount of *sigH*_Lsa _and *comEA *transcripts (Table 1). In contrast, induction of the P_atkY_-controlled copy of *sigH*_Lsa _led to a ~40-fold effective increase of *sigH *transcripts after 1 h, and ~ 200-fold after 4 h. *comEA *transcript levels were highly increased (over 300-fold), but only when *sigH*_Lsa _was 40 fold over-expressed (a 20-fold increase of *sigH*_Lsa _mRNA did not alter *comEA *expression, Table 1). The need for high *sigH*_Lsa _overexpression may indicate the need to overcome posttranscriptional controls to produce enough active σ_Lsa_^H^. This proposal is supported by observations in *B. subtilis*, where σ_Bsu_^H ^was shown to be subjected to multiple controls [5,29], and in the genus *Streptococcus*, where high levels of ComX overexpression were required to artificially induce competence [30], likely due to the negative control of ComX stability by a Clp protease complex [30,31].

**Table 1:** Relative expression ratio^$ ^of *sigH *and *comEA *with or without overexpression of *sigH*

Sample	*sigH*(wt)* i		*sigH*(hy)* ni		*sigH*(hy)* i	
**Calibrator**	*sigH*(wt)* ni		*sigH*(wt)* ni		*sigH*(wt)* i	

**Measured effect**	**control of the inducer effect in wt strain**		**presence of the additional copy of *sigH***		**cumulative effect of induced additional copy**	

Time (h)	1	4	1	4	1	4

*sigH*	1	1	7	24	40	200

*comEA*	1	1	2	3	370	80

^$ ^Expressed as fold change of transcripts amounts of each gene in each given sample relative to the indicated calibrator and normalized with *ldh*. Results are the mean of two independent experiments. The level of *ldh *transcripts was stable, irrespective of the copy number or induction status of *sigH *(e.g. mean fold change across all induced samples relative to non induced samples: 0.9 ± 0.2). Note that *sigH *is present at one (chromosomal) copy in *sigH*(wt)* and at two copies (one additional copper-controlled copy on a plasmid) in *sigH*(hy)*; the transcription of both is measured simultaneously. ni and i refer to 'not induced' and 'induced', respectively.

*comEA *transcription was not increased at the onset of stationary phase in the WT nor in the induced *sigH*(hy)* strain, suggesting that the competence genes are not naturally induced under laboratory conditions. Activation of *comEA *tended to diminish after a four hour-induction despite high levels of *sigH*_Lsa _transcripts, possibly indicative of another regulatory loop on *comEA *or post-transcriptional regulation of *sigH*_Lsa_. This transcription pattern was similar for *comGA *exhibiting a 280-fold increase in transcript amounts one hour after *sigH*_Lsa _induction in *sigH*(hy)* followed by a 3-fold decrease between one and four hours. These results show that in *L. sakei*, conditions of σ_Lsa_^H ^overexpression lead to activation of *comEA *and *comGA*. Nevertheless, other factors likely modulate *com *gene expression, as suggested from the drop of expression late in growth.

### Global transcriptome profiling of the σ^H ^overexpression strain suggests the existence of a genetic competence state

To identify the genes of *L. sakei *regulated by σ_Lsa_^H^, the experimental system described above was used in a full-genome comparative transcriptome analysis of *sigH*(hy)* and *sigH*(wt)* after one hour induction with 30 μM CuSO_4_. Quantification and statistical analysis of the microarray data (see Methods for parameters) led to relatively few differentially expressed candidate genes. The overexpressed *sigH *gene in *sigH*(hy)* was 11 ± 3 times induced compared to the WT strain in this microarray experiment; qPCR-based quantification of the same RNA samples showed a 149 ± 42-fold greater expression relative to the WT strain, confirming the successful overexpression of *sigH*_Lsa_. Differences in fold ratios between microarray-profiling and qPCR analysis are not unusual but were high in our experiment; they might reflect a less efficient detection on microarray or an overestimation by qPCR especially when genes are weakly expressed in one of the conditions, which seemed to be the case for the *com *genes.

Based on statistical tests (P value < 0.05), our microarray analysis initially identified some 25 candidate genes whose expression was likely affected by *sigH*_Lsa _overexpression; behavior of several genes was confirmed by qPCR (Table 2). The known genes can be grouped into two main functional categories: competence (DNA uptake) and DNA metabolism. All the late competence (*com*) operons encoding structural elements of the DNA uptake machinery were highly activated by *sigH*_Lsa _overexpression. In contrast, transcription of *ssb*, regulated as a late competence gene in *B. subtilis *[32], was nearly constant or only very weakly induced. Other genes involved in DNA metabolism, and known to be induced during the competence state in other species, i.e., recombination genes *recA *and *dprA*, both of which are involved in natural bacterial transformation in different species [33], gave a contrasted picture when their transcription was specifically measured by qPCR. Whereas *recA *was little activated, expression of *dprA *was highly induced in the *sigH*(hy)* context (Table 2).

**Table 2:** Genome-wide transcriptome profiling of SigH_Lsa _overexpression in *L.sakei *23 K

Functional category and CDS	Gene Name	Product	Pvalue (Bonferroni) common variance model	Pvalue (FDR) varmixt model	Expression *sigH*(hy)*/	ratio$ *sigH*(wt)*
					
					microarray	qPCR
**Competence**						
LSA0492	comFA	DNA uptake machinery §	1.54E-02	> threshold	1.5 ± 0.4	286 ± 88
LSA0493	comFC	DNA uptake machinery	0	3.56E-03	2.2 ± 0.2	
LSA1069	comEC	DNA uptake machinery	9.52E-10	1.31E-02	1.9 ± 0.2	
LSA1071	comEA	DNA uptake machinery	0	7.23E-03	2.5 ± 0.3	261 ± 115
LSA1301	comGF	DNA uptake machinery	0	2.71E-04	3 ± 2	
LSA1302	comGE	DNA uptake machinery	0	1.44E-06	3.7 ± 0.5	
LSA1303	comGD	DNA uptake machinery	0	2.21E-04	2.8 ± 0.3	
LSA1304	comGC	DNA uptake machinery	0	5.62E-12	7 ± 2	421 ± 104
LSA1305	comGB	DNA uptake machinery	1.02E-10	3.57E-02	2.0 ± 0.3	
LSA1306	comGA	DNA uptake machinery	3.17E-09	7.25E-03	1.9 ± 0.2	
LSA1771	comC	DNA uptake machinery	0	4.10E-06	3.2 ± 0.2	608 ± 199
**DNA metabolism: replication, repair, recombination, RM**						
LSA0008	ssb	Single-stranded DNA binding protein	> threshold	3.88E-02	1.4 ± 0.1	1.2 ± 0.3
LSA0146		Putative DNA methyltransferase (apparently stand-alone)	1.55E-04	> threshold	1.6 ± 0.4	
LSA1299		Putative DNA methyltransferase (apparently stand-alone)	2.48E-08	> threshold	1.9 ± 0.4	
LSA1338	exoA	Exodeoxyribonuclease III	1.36E-07	> threshold	1.8 ± 0.3	
**Purines, pyrimidines, nucleosides and nucleotides**						
LSA0533	iunh2	Inosine-uridine preferring nucleoside hydrolase	1.14E-05	> threshold	1.7 ± 0.4	
**Energy metabolism**						
LSA1298	ack2	Acetate kinase	4.27E-09	> threshold	1.9 ± 0.4	
**Translation**						
LSA0009	rpsR	Ribosomal protein	1.67E-02	> threshold	1.5 ± 0.4	
**Regulatory function**						
LSA0421		Putative transcriptional regulator, MerR family	0	3.56E-03	2.5 ± 0.5	
**Hypothetical protein**						
LSA0040		Hypothetical protein, conserved in some lactobacilli	0	3.56E-03	2.5 ± 0.5	
LSA0409		Hypothetical integral membrane protein	3.02E-05	7.25E-03	0.61 ± 0.01	
LSA0536		Hypothetical protein with putative NAD-binding domain, NmrA structural superfamily	6.28E-06	3.32E-02	1.6 ± 0.4	
LSA0779		Hypothetical protein, peptidase S66 superfamily	4.77E-05	> threshold	0.6 ± 0.1	
LSA0991		Hypothetical protein with putative NAD-binding domain, NmrA structural superfamily	1.02E-04	> threshold	1.6 ± 0.2	
LSA1475		Hypothetical protein, conserved in bacteria	1.62-12	> threshold	2.1 ± 0.5	

CDS £	Gene Name	Product				qPCR

LSA0487	recA	DNA recombinase A				2.7 ± 0.7
LSA0992	dprA	DNA protecting protein, involved in DNA transformation				2163 ± 1242

$ Expression ratios represent the fold change in amounts of transcripts in the strain overexpressing SigH relative to the WT control strain. For the microarray experiment they were calculated from log_2_ratio; for the qPCR they were calculated by the 2^-ΔΔCt ^method described in Methods. Genes underexpressed in the context of SigH overexpression have a ratio < 1. Standard deviation is indicated (weak accuracy for qPCR experiments may be due to Ct at the detection limit for basal level).

§ see additional file 3: Competence DNA uptake machinery of *B. subtilis *and comparison with *L. sakei*.

£ not found statistically differentially expressed in the microarray transcriptome experiment, checked by qPCR.

Two genes coding for hypothetical proteins, LSA0409 and LSA0779, were down-regulated in the *sigH*_Lsa _overexpression strain. As sigma factors are usually positive regulators, we consider it likely that down-regulation of these genes is an indirect effect of *sigH*_Lsa _overexpression, e.g., this effect could correspond to σ^H^-mediated activation of an unidentified repressor. The sole transcriptional regulator (LSA0421) listed as σ^H^-activated in Table 2 is probably not responsible for this effect, since MerR-type regulators reportedly act as activators [34]. No clue for the function of LSA0421 could be drawn from the genetic context or multiple alignments with other MerR-type proteins from *E. coli *and *B. subtilis*.

Overall, the qPCR analysis validates our statistical analysis of the microarray data based on the common variance model associated with the correction of Bonferroni (Table 2). Indeed, although calculated expression ratios were very similar for *comFA *and *ssb *(around 1.5), the former, which had an associated P value < 0.05 with the Bonferroni correction, was confirmed as overexpressed in the qPCR analysis, whereas the latter (which passed the other applied statistical test) was found to be almost unaffected by σ^H ^in the qPCR analysis (Table 2). Therefore, we expect that all genes with a better score than *comFA *in the microarray anlaysis (i.e. P value Bonferroni ≤ 1.54 E-02) are good candidates for belonging to the σ_Lsa_^H ^regulon. Altogether, results of this study thus identify 25 genes as belonging to the σ_Lsa_^H ^regulon. Some genes (e.g., *dprA*), while truly activated by σ_Lsa_^H^, may not be detected in this microarray experiment, indicating the need for further studies to define the full regulon.

Transcriptional reprogramming caused by *sigH*_Lsa _overexpression is consistent with the existence of a competent state in *L. sakei*, supported by the observed up-regulation of *com *genes involved in pseudopilus morphogenesis and DNA translocation as well as of *dprA *(which shares 47% aa identity with the *S. pneumoniae dprA *gene product). *ssb *and *recA *appear little or not activated one hour after *sigH*_Lsa _induced overexpression, whereas their level of induction during the competence state in *S. pneumoniae *and *B. subtilis *reportedly varies from 5 to over ten-fold [32,35]. These genes might be transiently regulated (in a narrower window than *com *operons and *dprA*), regulated by other factors, or their up-regulation may not be required in *L. sakei*. Indeed both genes participate in the bacterial vegetative life cycle and are expected to be transcribed at a significant basal level when cells are not in the competence state [36]. Interestingly, *L. sakei *possesses a unique *ssb *gene (*ssbA*-type), whereas *B. subtilis *and *S. pneumoniae *have paralog genes [36,37]. The need for a transformation-dedicated SSB protein has been discussed [37]. Although known natural transformation is frequently associated with multiple *ssb *in Firmicutes [37], an additional competence-induced SSB may be a facilitator rather than an essential contributor to the transformation process, since transformation frequency is only reduced by two- to ten-fold when *ssbB *is inactivated in *S. pneumoniae *or in *B. subtilis *[36].

### Is *L. sakei *capable of natural genetic transformation?

As the σ^H^-activated transcriptome of *L. sakei *was indicative of a competence state, we looked for genetic transformation in this species. The first strategy involved the overproducer strain *sigH*(hy)*. As the strain harbors a resident plasmid conferring erythromycin resistance, a compatible plasmid conferring chloramphenicol resistance (pVI1056) was used for transformation tests. After cultivation under inducing conditions (i.e., addition of 30 μM CuSO_4_), the strain was mixed with 100 ng of pVI1056 and plated on selective medium. Experiments were performed under various conditions: i) glucose concentration at 1% or 0.1%, ii) growth in microaerobiosis or aeration, and induction at early, middle or late exponential phase iii) addition of MgCl_2 _(80 mM) during contact between cells and DNA, after middle phase induction in microaerobiosis or aeration; in addition, chromosomal *L. sakei *DNA (1 μg) was also used as exogenous DNA. None of the tested conditions resulted in DNA transformation.

Development of natural transformation may be strain-dependent [30,38,39]. We therefore used a second strategy (independent of *sigH *overexpression) to test different *L. sakei *isolates for competence, using a protocol where DNA and strains are deposited on solid medium. In addition to 23 K, four strains (64 K, 332 F, 160 K and LTH675) were chosen based on their different genotypes and genome sizes, and known capacities to be transformed by electroporation [20,58]; Chaillou and Anba, personal communication]. Two replicative plasmids and chromosomal *L. sakei *DNA were used. In spite of varying media (MRS or MCD) and incubation temperatures (4°C, 30°C or 37°C), no colonies were recovered on selective medium.

Among the *Lactobacillales*, natural genetic transformation has been reported for many species of the genus *Streptococcus *[40] and has been suspected for one *Lactobacillus *[41]. In recent years, natural transformation has been demonstrated in several Gram-positive or Gram-negative species, previously unsuspected to develop genetic competence [42,43]. Overproduction of the activator protein has proven to be an efficient way to trigger genetic transformation in various bacteria, e.g., TfoX in *Vibrio cholera*e [42] or ComK in *Bacillus *species [14,44]. However, artificially raising transcription of the ComX master regulator gene initially failed to induce efficient genetic transformation for *S. thermophilus *strain LMD-9 [30], which was very recently shown to be efficiently naturally transformable [37]. In the present and previous studies, a failure to achieve a competent state in bacteria (either spontaneously or triggered by artificial overexpression of a master activator) may be due to the use of inappropriate growth conditions, which might not allow the detection by the cells of a needed specific triggering factor [38,42] or the full activation of multiple converging regulatory pathways [30]. As such, in the case of *L. lactis *[21], *S. pyogenes *[45], *S. aureus *[12], or *L. sakei *(this paper), only the activation of several competence genes, but not genetic transformation, could be obtained after ectopic expression of the activating sigma factor. Our results suggest that some of the genes induced in other naturally competent Firmicutes are not activated by the sole *sigH*_Lsa _overexpression in *L. sakei*. In the case of *S. pyogenes*, the identification of a novel pheromone in related species of *Streptococcus *might pave the way for deciphering a natural genetic transformation system in this bacterium [46]. Whether competence gene activation by ComX/σ^H ^is linked to the capacity of being transformable in these species, and under which conditions, remains to be determined.

### Effect of *sigH *on *L. sakei *survival

**Figure 5:**
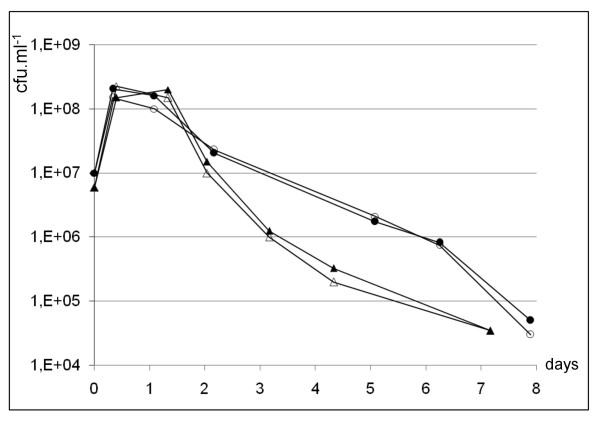
**Effect of overexpression or deletion of *sigH *on viability of *L. sakei *in mixed cultures with WT strain**. Each pair of mutant and WT strains has been mixed after separate growth until an OD_600 _of 0.3, in MCD medium at 30°C in microaerobiosis. Enumeration on appropriate agar plates allowed to distinguish WT from mutant strains. *sigH*(nul) mutant (black triangles) was mixed with WT strain 23 K (empty triangles). *sigH*(hy)* overexpression mutant (black circles) was mixed with *sigH*(wt)* strain (empty circles), and 30 μM CuSO_4 _was added to the culture. Curves are the mean of two independent experiments.

**Figure 6:**
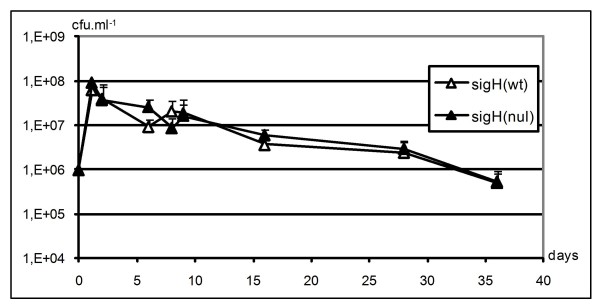
**Long-term viability of *L. sakei *in meat juice at 30°C**. Curves are the mean of three independent experiments; error bars represent standard deviation (logarithmic scale).

No indication of another large adaptive response triggered by σ_Lsa_^H ^could be deduced from the few other up-regulated genes distributed in different functional categories. We also searched for phenotypic effects linked to a putative role of σ^H ^on survival in stationary phase or after DNA damage. For that purpose, we constructed a *sigH*(nul) null mutant (see Methods) and compared the effect of overexpression or absence of σ_Lsa_^H ^relative to WT strains on growth and stationary phase survival in MCD medium under aerobiosis, microaerobiosis or anaerobiosis, as well as on UV resistance. No changes in any of the above tests could be attributed to σ^H ^expression levels under the conditions tested (data not shown). Interestingly, all the strains revealed UV resistance, since the fraction of each population killed by 254 nm irradiation was in the range of 0-5% at 60 J.m^-2^, 60-70% at 80 J.m^-2^, 95-98% at 100 J.m^-2 ^and 99.5-99.9% at 120 J.m^-2^. This is to be compared to the reported 100% killing of *Lactobacillus brevis *exposed to 254 nm UV light at 70 J.m^-2 ^[47]. Competition experiments in mixed cultures revealed no imbalance in growth or survival between the σ^H ^overproducing or σ^H ^deficient and WT strains in MCD medium (Figure 5). As MCD medium may not represent a usual environment for the bacterium, a meat-derived medium was tested for comparison of *sigH*(nul) and WT strains. *L. sakei *showed prolonged stationary phase survival in meat juice, where about one percent of the population was still alive after one month at 30°C (Figure 6). Inactivation of *sigH *brought no striking change to the phenotype.

## Conclusions

This study gives further insight into the function of σ^H^-family sigma factors from Firmicutes, whether they belong to sporulating or non-sporulating bacteria. Two main models emerge: ComX of non-sporulating streptoccoci is the master activator of the genetic competence pathway, participating directly in the transcription of late *com *genes [15]. σ^H ^of *B. subtilis *activates a complex response leading to spore formation as an ultimate outcome and to the development of genetic competence during a transition period. Unlike ComX, σ_Bsu_^H ^does not directly activate genes encoding the DNA uptake machinery, but participates as an intermediate in the upstream signaling pathway controlling the master regulator of competence ComK [5,48]. *sigH *genes from the non-sporulating *L. sakei *and *S. aureus *species are organized similarly to the *sigH *locus of the sporulating bacterium *B. subtilis*. However, unlike *B. subtilis*, they act like streptococcal ComX by activating late *com *genes [[12]; this paper]. We speculate that this function may be conserved in the order *Lactobacillales*, irrespective of the exact location of the so-called ComX or σ^H ^encoding gene. The regulon of σ_Lsa_^H ^as deduced by assessing the effects of σ_Lsa_^H ^overexpression was rather small. It should be mentioned that the genome size of the model strain used was 136 kb less than the average size within the species [20] and that our strategy mainly identified genes that were strongly affected by σ_Lsa,_^H ^independently of possible other, undetermined, environmental signals. A large number of reported regulatory effects of σ_Bsu_^H ^are actually mediated in conjunction with other transcriptional regulators, especially Spo0A and AbrB [5]. *L. sakei *and more generally *Lactobacillales *do apparently not possess orthologs of these regulatory proteins, neither do they possess a ComK homologue. Deciphering all the functions of the conserved σ^H ^sigma factor in other groups of Firmicutes, sporulating or not, and equipped with different combinations of these known global regulators will probably help to clarify σ^H ^evolution in this group of bacteria.

## Methods

### Media and growth conditions

*L. sakei *was grown at 30°C in MRS medium [49] or in the chemically defined medium MCD [50], both containing 1% glucose. A two-step preculture was used to assure reproducibility of experiments. First, 5 ml MRS was inoculated with one freshly isolated colony and incubated for about 8 h without agitation. After centrifugation, cells were resuspended in MCD at an OD_600 _of 1 and 10 to 20 μl of the suspension was used to inoculate 40 ml of fresh MCD. This second preculture was incubated without agitation for about 15 h so as to collect the cells in exponential growth phase. This preculture was then concentrated to an OD_600 _of 10 in fresh MCD, and used to inoculate the test culture to give an initial OD_600 _of 0.1 to 0.15. Unless otherwise indicated, growth conditions under microaerobiosis were used. Different aeration conditions were obtained by varying the agitation parameter and volume of cultures. Typically microaerobiosis corresponded to non-agitated cultures in closed centrifugation tubes, aerobiosis to 140 rpm of 1/10^th ^full ehrlenmeyer flasks, and anaerobiosis to N_2_-saturated cultures in sealed bottles [51]. Growth was followed by OD_600 _measured in a Secomah spectrophotometer. As 30 μM CuSO_4 _may be added to the culture, we monitored its global effect on *L. sakei *growth. In static or anaerobic growth conditions, 30 μM CuSO_4 _had no effect on growth. In aeration conditions, 30 μM CuSO_4 _had a slight effect on growth (2-10% lower OD_600 _at the end of growth), and slightly extended viability.

Meat juice was obtained from beef meat homogenized with half volume of sterile water in a Stomacher for 2 cycles of 3 min each. The supernatant obtained after centrifugation (10,000*g *for 15 min) was filter sterilized and stocked at -20°C (M.-C. Champomier Vergès, unpublished). *Escherichia coli *(DH5αF' or TGI) was cultured aerobically in LB at 37°C. Selective pressure for plasmids was maintained in *E. coli *with ampicillin 100 mg.l^-1^, and in *L. sakei*, with erythromycin 5 mg.l^-1^.

### DNA techniques

Standard procedures were used for DNA manipulation. Classical PCR reactions were performed with Taq polymerase (Fermentas) or Pfu polymerase (Promega) for cloning purpose, and run in MJ research PTC-200 thermocycler. Extraction of plasmids and chromosomal DNA as well as electroporation of *L. sakei *and *L. casei *BL23 was carried out as described [52]. Primers are listed in additional file 4.

### Diversity of *sigH *in *L. sakei*

*L. sakei *strains (18, 21, 23 K, 64, 112, 160 K, 300, 332, JG3, MF2091, MF2092, ATCC15521, CIP105422, SF771, LTH677, LTH2070) were from our collection or different sources as described [20]. PCR amplification of the *sigH *locus was carried out with two pairs of primers (AML31/AML32 and AML50/AML58). Sequence of the 561 nt fragment corresponding to entire CDS and the 77 nucleotides of the upstream intergenic region was performed on PCR-amplified genomic DNA using each of the four primers. Pairwise distances were calculated by MEGA 4 [53] using a Kimura 2-parameter substitution model.

### Construction of *sigH *mutant and *sigH *expression strains

SigH production and *sigH *mutant strains were constructed from RV2002, a derivative of *L. sakei *23 K that had undergone a deletion of the *lacLM *gene encoding β-galactosidase [23]. Their construction used plasmids pRV610 and pRV613 [27] which contain two replication origins, one functional in *E. coli *(pBluescript) and one for Gram-positive bacteria (pRV500).

The *L. sakei *σ^H ^overproducer strain *sigH*(hy)* was obtained by introducing plasmid pRV619 into RV2002. pRV619 was constructed from pRV613 which bears the P_atkY _copper-inducible promoter cassette of *L. sakei *fused to the *E. coli lacZ *reporter gene [27]. *lacZ *was replaced by *sigH*_Lsa _in pRV619 as follows. The *sigH*_Lsa _coding region was PCR-amplified from *L. sakei *strain 23 K chromosomal DNA with primers AML31 and AML32 and the BamHI/XbaI fragment was cloned into pRV613 digested by the same enzymes, using *Lactobacillus casei *BL23 as a host, since neither *L. sakei *nor *E. coli *were successful for direct cloning. The extracted plasmid was then successfully introduced into *E. coli *from which we could obtain the needed high-quality plasmid preparation (Qiagen Plasmid Kits) to electroporate *L. sakei *RV2002.

The *L. sakei sigH *null mutant (RV7003 designated *sigH*(nul) in the text) was obtained via a double cross-over homologous recombination with the pRV622 integrative plasmid. To inactivate the *sigH *gene we deleted its putative promoter and the first 34 codons while introducing an in-frame stop codon at the endpoint of the deletion (see additional file 2: Genotypes of *L. sakei *strains affected in *sigH*). The upstream and downstream fragments were generated by PCR using respectively AML51/AML52 and AML53/AML54 primer pairs, thereby introducing an EcoRI site in *sigH*. Each amplicon was digested with EcoRI, followed by DNA ligation and digestion with PstI and XhoI. The resulting 1.1 kb fragment was then reamplified by the distal primers AML51 and AML54 and cloned by blunt-end ligation after treatment with T4 polymerase, into the pRV610 cloning vector [27] cut by SmaI. As above, *L. casei *BL23 was used as a host for cloning, giving plasmid pRV621. This plasmid was then successfully introduced into *E. coli *and an intra-molecular deletion of the Gram + replication cassette was generated between unique restriction sites EcoRV and KpnI repaired by T4 polymerase, giving pRV622 which replicates in *E. coli*. Gene replacement in *L. sakei *was carried out as described [23], with two successive single crossovers, the first one leading to chromosomal integration of the plasmid (maintained by erythromycin selection), and the second one allowing plasmid excision, monitored by loss of erythromycin resistance. The mutant chromosomal structure was checked by PCR. Correct sequence of the inserts was checked for pRV619 and pRV622.

### Induction of P_atkY _promoter utilization and monitoring using β-galactosidase activity

The copper-inducible P_atkY _promoter was used as described [27] for *sigH *overexpression. For this purpose, CuSO_4 _was added to a final concentration of 30 μM when cultures reached an OD_600 _of about 0.4. Induction of the P_atkY _promoter was controlled with the *sigH*(wt)* strain, harboring a P_atkY_-directed *lacZ *reporter gene. Sampling was done one hour after induction and β-galactosidase activity was measured according to [23] using ONPG (*o*-nitrophenyl-β-D-galactopyranoside) as a substrate. Activities expressed as Miller units relative to OD_600 _of the culture [23] were observed to be between 10 and 25 after induction, whereas the non induced standard was around 0.5.

### Extraction of total RNA

*L. sakei *strains were cultivated at 30°C in MCD under microaerobiosis following the standardized procedure described in the upper section, in the presence of erythromycin for plasmid-containing strains. Cultures of *L. sakei *were distributed in as many centrifugation tubes as scheduled collecting points and were incubated at 30°C without agitation. Cell pellets were collected by brief centrifugation at the time indicated, freezed in liquid nitrogen and preserved at -80°C. Total RNA was extracted with TRIzol reagent (Invitrogen) as previously described [54]. Integrity of RNA was checked by Bioanalyzer 2100 (Agilent). RIN values were above 9.

### Whole-genome microarray analysis

The *L. sakei *microarray http://migale.jouy.inra.fr/sakei/?q=supplement comprises all the identified coding genes of strain 23 K represented by 70 nt long oligonucleotides synthesized by Operon Biotechnologies Inc. The manufacture of DNA chips as well as labelling, hybridization and image analysis were performed at the Biochips platform of Toulouse-Genopole http://biopuce.insa-toulouse.fr/Maquette/en/. Each oligonucleotide was spotted in triplicate on UltraGaps coated slides (Corning^® ^Life Sciences). Total RNA (5 μg) was reverse transcribed and labeled with either Cy5 dCTP or Cy3 dCTP (Amersham Biosciences) using the ChipShot™ Direct Labeling System (Promega). Labelled cDNA (50 pmol of Cy3 and 50 pmol of Cy5) was included in a dye-switch hybridization protocol carried out in an automatic hybridization chamber (Discovery, Ventana Medical system). Images of scanned slides (GenePix 4000A Scanner-Axon Instruments) were analyzed, spots delimitated and hybridization signals were quantified and transformed into numerical values by GenePixPro v.3.01 software (Axon). Background noise was rather homogeneously distributed and only a few spots were saturated at 75%, mainly those corresponding to rRNA.

Statistical analysis of the data was conducted with the R Package Anapuce 2.1 by J. Aubert http://www.agroparistech.fr/mia/doku.php?id=productions:logiciels. Normalization rested on a global lowess regression followed by a block correction, after filtering out spots with a signal to noise ratio < 3 (including empty spots). Background was not subtracted. Differential analysis was performed on average values for the triplicate spots obtained by the MeanBySpot function. Three models of variance were applied: one variance by gene, a common variance for all the genes and clusters of genes with equal variance (varmixt). Two different multiple testing corrections were used to adjust raw P-values, Bonferroni correction (which is the most stringent) and False Discovery Rate of Benjamini and Hochberg, with a nominal type I error rate set to 0.05.

### Microarray accession numbers

The microarray data have been deposited in the Array Express database http://www.ebi.ac.uk/arrayexpress/ under the accession numbers A-MEXP-2068 (array design) and E-MEXP-3238 (experiment).

### Real-time qPCR for quantitation of steady-states transcripts

The mRNAs corresponding to the genes of interest were measured by qPCR using SYBR Green fluorescence, appropriate specific primers (see additional file 4: list of primers) and total first-strand cDNA as template. Contaminating DNA was first eliminated from RNA samples using TurboDNA-free from Ambion. Two series of reverse transcription and qPCR experiments were carried out in this study, for monitoring temporal expression and overexpression of *sigH *on the one hand and for validation of microarray data on the other hand. Total first strand cDNA was produced with random hexamer primers (Random Primer 6 5'd(N6)3', Biolabs) using either PowerScript Reverse Transcriptase (Clonetech) or PrimeScript Reverse Transcriptase (Takara). The quality of each template cDNA was checked using the Bioanalyzer 2100 (Agilent). qPCR was performed using specific primers (75-100 nM each) according to the recommended protocol for each SYBR Green mix used (SYBR Green MasterMix 2X from ABgene or MESA GREEN MasterMix from Eurogentec). Reactions were run on an ABI PRISM 7900 HT instrument (Applied Biosystems) or a Mastercycler Realplex 2 S instrument (Eppendorf) using 40 cycles of denaturation at 95°C for 15 s and extension at 60°C for 1 min. The cycles were preceded by DNA polymerase activation at 95°C and followed by a denaturation cycle to check the specificity of the PCR products. Mean Ct obtained for studied genes were between 16 and 28.5, with the exception of *comC *and *dprA *in WT strain at 31 and 32.9 respectively (in the same time 'No Template Controls' gave no signal after 34 cycles).

Primers were designed with Primer Express 2 (Applied Biosystems) or Primer 3 http://frodo.wi.mit.edu/primer3 and validated by determining slopes of standard curves for PCR efficiencies between 90% and 100%. In this context, we used the 2^-ΔΔCt ^method to express results as fold change in the expression of each gene of interest relative to a calibrator sample and a reference gene used as an internal control for normalization of the results [55]. The stability of transcription of the chosen reference gene *ldh *was checked by standard curves performed for all environmental conditions used in this study. Unless otherwise indicated, quantitation experiments were performed with three independent samples, each well being duplicated two or three times. Values are expressed as mean ± standard deviation.

### Viability and UV assays

Viable bacteria were counted by plating serial dilutions on MRS agar and incubating at 30°C for one to four days. For mixed cultures, classical enumeration on MRS supplemented with Xgal (5-bromo-4-chloro-3-indolyl-β-D-galactopyranoside, 0.04 g.l^-1^) distinguished *sigH*(hy)* (white) from *sigH*(wt)* (blue) as well as *sigH*(nul) (white) from 23 K *lacLM *+ (blue). For other tests, sampling for stationary phase survival in MCD was done after 6-8 hour culturing which corresponds to growth arrest, then once or twice a day. In these cases, comparative enumeration was performed by depositing drops (5 μl) of serial decimal dilutions for each strain on an agar plate. UV resistance was examined by exposing bacteria freshly plated on MRS medium to 254 nm UV-light (VL-15 C, Apelex) with fluences of 40 to 120 J/m^2 ^(by step of 20) measured by the radiometer VLX-3 W equipped with a 254 nm sensor (Vilber Lourmat, France). For that purpose, drops (5 μl) of serial dilutions of bacterial suspensions were rapidly plated after growth in MCD medium, one hour after addition of 30 μM CuSO_4 _or at the same time without addition of CuSO_4_.

### Natural genetic transformation

Exogenous DNA used in this study comes from plasmid (pVI1056, pRV620, and pGKV259) and *L. sakei *chromosome (strain RV2000), and confers resistance to chloramphenicol (10 mg.l^-1^) to recipient bacterium. RV2000 is a derivative of *L. sakei *23 K in which the *cat*194 gene interrupts *ldh *[56]. pVI1056 (Van de Guchte in [27]) is a 7.5 kb pIP501-derived shuttle plasmid (theta-replicating), known to be replicative in *L. sakei*. pRV620 is a 5.6 kb shuttle plasmid derived from theta-replicating plasmid pRV500 of *L. sakei *[27]. pGKV259 is a broad host range 5 kb rolling circle plasmid [57]. Plasmids were purified from *E. coli *TG1 using Qiagen Plasmid preparation Kits, and checked by electrophoresis on agarose gel for the presence of multimers. 10 ng of plasmids pVI1056 and pRV620 were reportedly able to transform *B. subtilis *naturally competent cells [27]. For transformation tests with *L. sakei*, *sigH*(hy)* overexpression strain was cultivated in MCD as described above. After 30 to 60 min induction with 30 μM CuSO_4 _(at usual OD_600 _of 0.4 and at OD_600 _of 0.2 and 0.9 when indicated), aliquots of 100 μl of cell suspension were mixed with 100 ng pVI1056 DNA in a microtube and incubated for one hour at 30°C. Suspensions were then plated on selective MRS medium and incubated for several days at 30°C. As *sigH*(hy)* strain already contained a plasmid, its transformability with incoming plasmid was verified by electroporation. Transformation tests on plates with other *L. sakei *strains were done as follows. 23 K, 64 K [plasmid-cured 64], 332 F [pRV500-cured 332], 160 K and LTH675 [20,52,58] were cultivated in liquid MRS and MCD medium until late exponential phase and plated on the same solid medium supplemented with 10 mg.l^-1 ^chloramphenicol. Drops of pRV620 and pGKV259 (50 ng each), and RV2000 chromosome (500 ng) were deposited on the plates which were then incubated at the indicated temperatures.

## Authors' contributions

SS participated in the design of the study, participated in the sequence alignments, carried out construction and characterization of overexpression strain and carried out part of the qPCR analysis. CB participated in the design of the qPCR analysis. AMCLC conceived and participated in the design of the study, carried out and supervised the rest experiments, and wrote the manuscript. All authors read and approved the final manuscript.

## Supplementary Material

Additional file 1 **Alignment of four σ^H^-group sigma factors**. Click here for file

Additional file 2 **Genotype of *L. sakei *strains affected in *sigH***. Click here for file

Additional file 3 **Competence DNA uptake machinery of *B. subtilis *and comparison with *L. sakei***. Click here for file

Additional file 4 **List of primers**. Click here for file
