# Supplementary material for: Alternative sigma factor σH activates competence gene expression in Lactobacillus sakei
Source: BMC Microbiol. 2012 Mar 12;12:32. doi: 10.1186/1471-2180-12-32 (PMC3364868; doi:10.1186/1471-2180-12-32)
Supplement: Additional file 1 — Alignment of four σH-group sigma factors. [file 1471-2180-12-32-S1.PDF]

Additional file 1 - Alignment of four σ^H-group sigma factors.

```
                                                              2
                                                      2.2                   2.4
Lsa-SigH    1   MEQTK-----------RDELLISAVAQNDSQALDVLFKAYLPMVYHTLAPYYIRLFDEDDWLQEARIVCYETCQCYD--CQKGKSFGSFYKLRFKNHILNLLRRELAQKRR
Bsu-SigH    1 MNLQNNKGKFNKEQFCQLEDEQVIEKVHVGDSDALDYLITKYRNFVRAKARSYFLIGADREDIVQEGMIGLYKSIRDFK--EDKLTSFKAFAELCITRQIITAIKTATRQKHI
Spn-comX    1                                  MIKELYEEVQGTVYKCRNEYYLHLWELSDWDQEGMLCLHELISREEGLVDDIPRLRKYFKTKFRNRILDYIRKQESQKRR
Sau-SigH    1 MKYDLTT------------QDSTIKRNNSINDKDFEKLVMDLQPLIIRRIKTFGFNHYDLEDLYQEILIRMYRSVQTFD--FSGEQPFTNYVQCLITSVKYDYLRKYLATNKR

                                                        4
                                                                  4.2
Lsa-SigH  100 -----GDRQAVLIAERPER---DAIS-------EDTVTYYQSQFELRCYL-ETLSPFELVSFRVLTGQLSIDEACCQLDCTIDQLK-RGQSRCQTKLKRYYQENR      186
Bsu-SigH  112 PLNSYASLDKPIFDEESDRTLLDVISGAKTLNPEEMIINQEEFDDIEMKMGELLSDLERKVLVLYLDGRSYQEISDELNRHVKSID-NALQRVKRKLEKYLEIREISL  218
Spn-comX   80 ----YDKEPYEEVGEISHR-----------------ISEGGLWLDDYYLFHETLRDYR---------NKQSKEKQEELERVLSNERFRGRQRVLRDLRIVFKEFTIRTH 159
Sau-SigH  100 -MDNLINEYRVTYPCAIKR-----------FDVENNYLNKLAIKELICQF-KYLSAFEKDVMYLMCEQYKPREIAQLMHVKEKVIY-NAIQRCKNKIKRYFKMI       189
```

Sequence from *L. sakei* 23K (Lsa) was aligned to those of *B. subtilis* 168 (Bsu), *S. pneumoniae* R6 (Spn) and *S. aureus* N315 (Sau) with clustalX [58] in the order of Blast results (http://blast.ncbi.nlm.nih.gov/Blast.cgi), showing identical (black background) and conserved (grey background) residues in at least 2 sequences (BoxShade Server, http://www.ch.embnet.org/software/BOX_form.html). Regions 2 and 4 as well as functional sub-regions predicted in σ^H_{Bsu} by sequence and structure conservation with primary sigma factors of *E. coli* and *B. subtilis* are indicated above the sequences [3]. Regions 2.4 and 4.2 were proposed to be involved in -10 and -35 recognition, respectively, and region 2.2 to form one of the primary interface with RNA polymerase [1, 3]. Except for a possible GAAT motif in the -10 region, promoter consensus sequences determined for σ^H_{Bsu} [5], σ^H_{Sau} [12], and ComX [34] are rather different (and sometimes degenerate), which may explain relative poor conservation across the cognate domains of sigma factors. Amino acid % identity between *L. sakei* and other sequences was calculated using clustalX.
