# Supplementary material for: Alternative sigma factor σH activates competence gene expression in Lactobacillus sakei
Source: BMC Microbiol. 2012 Mar 12;12:32. doi: 10.1186/1471-2180-12-32 (PMC3364868; doi:10.1186/1471-2180-12-32)
Supplement: Additional file 2 — Genotype of L. sakei strains affected in sigH. [file 1471-2180-12-32-S2.PDF]

Additional file 2 - Genotype of *L. sakei* strains affected in *sigH*

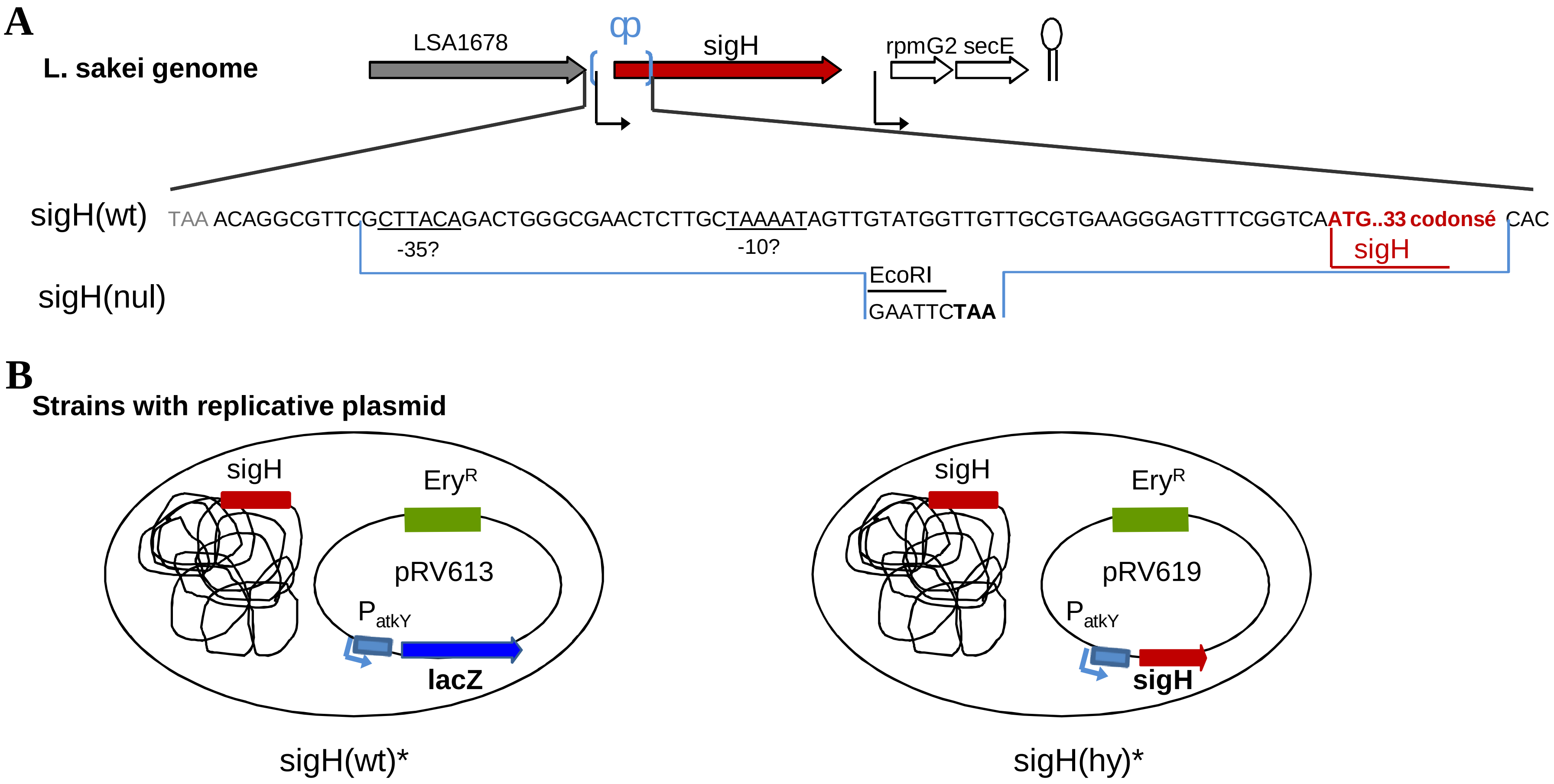

A. Features of the *sigH* locus in WT strain and null mutant. CDSs (open arrows), predicted promoters (broken arrows) and terminator (loop) are shown. Deletion in the null mutant is indicated inside brackets. Introduced stop codon is in bold type.

B. Schematic representation of the *sigH* overexpressing mutant *sigH*(hy)* and corresponding WT strain showing genes of interest on chromosome and additional plasmids. The copper-inducible promoter cassette is indicated as P_{atkY}
