# Supplementary material for: Alternative sigma factor σH activates competence gene expression in Lactobacillus sakei
Source: BMC Microbiol. 2012 Mar 12;12:32. doi: 10.1186/1471-2180-12-32 (PMC3364868; doi:10.1186/1471-2180-12-32)
Supplement: Additional file 3 — Competence DNA uptake machinery of B. subtilis and comparison with L. sakei. [file 1471-2180-12-32-S3.PDF]

Additional file 3 - Competence DNA uptake machinery of *B. subtilis* and comparison with *L. sakei*.

A

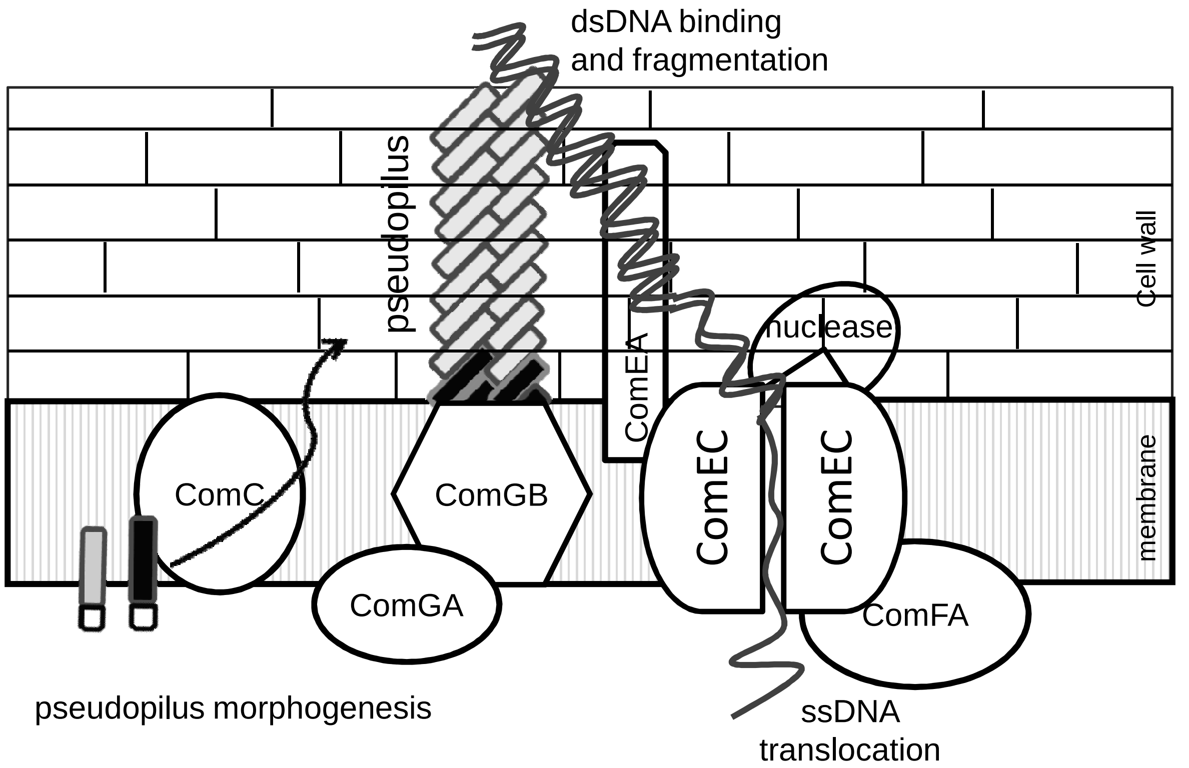

B

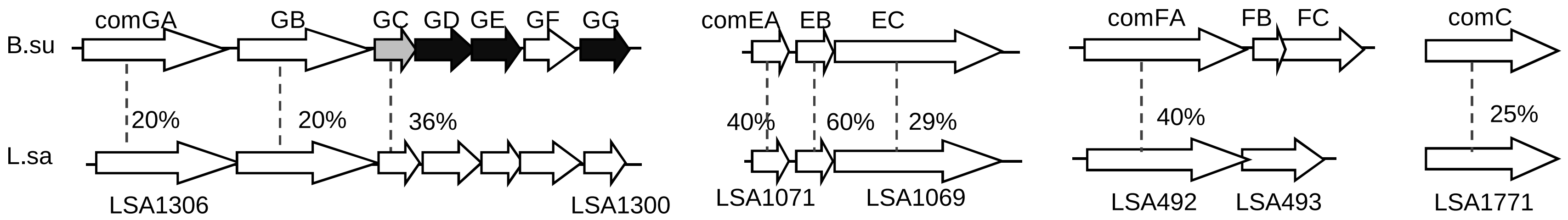

A. Schematic representation of DNA uptake machinery, modified from Chen and Dubnau [27]. The *B. subtilis* pseudopilus is constituted of pseudopilins, one major protein (ComGC, in grey) and several minor proteins (ComGD,GE,GF, in black), which undergo maturation and exported via a type IV leader peptidase (ComC). Its assembly requires the membrane protein ComGB and the trafficking NTPase ComGA. The pseudopilus serves the purpose of bringing DNA across the cell-wall to the translocation machinery, composed of a membrane-bound receptor (comEA), a pore (ComEC), an ATP-binding protein (ComFA), and an unidentified nuclease which degrades one strand of the incoming double-stranded DNA.

B. Comparative organization of the four late *com* operons in *B. subtilis* and *L. sakei*. Deduced amino acid % identity between some of the CDSs is indicated.
