# Supplementary material for: Alternative sigma factor σH activates competence gene expression in Lactobacillus sakei
Source: BMC Microbiol. 2012 Mar 12;12:32. doi: 10.1186/1471-2180-12-32 (PMC3364868; doi:10.1186/1471-2180-12-32)
Supplement: Additional file 4 — List of primers. [file 1471-2180-12-32-S4.PDF]

**Additional file 4   List of primers**

**sigH locus**

| Primer | Sequence (5' > 3') | Restriction site |
|---|---|---|
| AML31 | atggatccaGTGAAGGGAGTTTCGGTC | BamHI |
| AML32 | attctagaTTTGGTTAACGATTCTCTTG | XbaI |
| AML50 | AATCATCTTCATCCTCCTATG | |
| AML51 | atctcgaGTCAACCTTTGGTATAGCTG | XhoI |
| AML52 | atgaattCGAACGCCTGTTTAATCA | EcoRI |
| AML53 | atgaattcTAaCACACCCTGGCGCCAT | EcoRI |
| AML54 | aatctgcaGTCAATCTTAGAATCCAT | PstI |
| AML58 | atggatCCATGACGCTAAACGTTAT | |

**qPCR**

| Gene | Sequence of forward primer (5' > 3') | Sequence of reverse primer (5' > 3') |
|---|---|---|
| comC | CCATTATCACACCACGTTCG | GGCAGTGGCCACGTAAGTAT |
| comEA | GGTGGTCTACCCGGGAATTT | TGCATTTTTGAGCACCTCAAA |
| comFA | CAACGTTGTCAGCAAGTGGT | CCCTCTACCAAACGACCAAA |
| comGA | GGTCGCGCGCTTATTAGATC | GGTAGGCAATTCCCGTTAGACA |
| comGC | CGACGAGATTGGTCATCAAA | CCTTTGCGTGCTTTAATTGG |
| dprA | CAGCCCGCAATAAAATCATC | GGCCAAGTTAGCGGTAATCA |
| gyrA | CGACCAATATTCCACCGCATA | TGTTGCATCAGGATTATCCATCA |
| ldh | GCAAACATTGAAAAAGATCACCAA | GCATAAGCGTAGCTAGAACCAACA |
| recA | TCGATTCAGTTGCTGCTTTG | TGACATCAAACGGGCTTGTA |
| ssb | ATGGCGCAAGTCAGAACTTT | GAGTGTTATTTGCGGCTGGT |
| sigA | GCCGTGGCATGCAATTC | GATCAAATTTTTCAACAGCCTTCA |
| sigH | CCGGGCAGCTTTCAATTG | GCCCCGTTTCAACTGATCA |

The list of primers is divided into two sections according to the type of use in this study. Residues not matching the target sequence are indicated by small letters. Product length for qPCR analysis was in the range 68 to 110 nucleotides.
